# Anticancer Mechanisms of Bioactive Compounds from Sweet Potato (*Ipomoea batatas* L.) Leaves: A Systematic Review

**DOI:** 10.3390/foods15010093

**Published:** 2025-12-29

**Authors:** Saleh Shafique Chowdhury, Muhammad Abul Kalam Azad, Nanziba Ibnat, Shahidul Islam

**Affiliations:** Department of Agriculture/Agricultural Regulations, University of Arkansas at Pine Bluff, 1200 North University Dr., 148 Woodard Hall, Mail Slot 4913, Pine Bluff, AR 71601, USA

**Keywords:** sweet potato leaves (SPL), *Ipomoea batatas*, apoptosis, bioactive compounds, cancer, anticancer mechanism, phytochemicals

## Abstract

Sweet potato leaves (SPL) are increasingly recognized as a significant source of nutritionally and pharmacologically important bioactive compounds. This systematic review critically synthesizes current in vitro, in vivo, and preclinical data to evaluate the cancer preventive properties of SPL, with emphasis on their phytochemical composition, molecular mechanisms, and therapeutic relevance. A comprehensive literature search across major scientific databases (2015–2025), guided by PRISMA methodology, initially identified 29,416 records. After applying pre-specified inclusion and exclusion criteria and screening titles, abstracts, and full-texts, 38 eligible studies were included. The compiled evidence demonstrates that SPL contains high concentrations of phenolic acids, flavonoids, peptides, carotenoids, and dietary fiber, all of which contribute to diverse anticancer activities. Reported mechanisms include apoptosis induction, cell-cycle arrest, limitation of tumor propagation and metastatic activity, regulation of oncogenic pathways (PI3K/Akt, MAPK, NF-κB), modulation of inflammatory mediators, and suppression of angiogenesis. These effects were observed across multiple cancer models, including liver, colon, breast, lung, and prostate cancers. In addition, SPL represents a promising natural source of anticancer agents, significant gaps remain, particularly regarding standardized extraction procedures, phytochemical characterization, bioavailability, and human clinical validation. Overall, this review underscores SPL as a sustainable and underutilized plant resource with potential applications in functional foods, nutraceuticals, and adjunctive cancer therapy, while highlighting the need for mechanistic studies, pharmacokinetic investigations, and well-designed clinical trials to support future translational development.

## 1. Introduction

Edible plants constitute a significant source of bioactive compounds with potential chemopreventive and therapeutic effects. Recently, extensive research has focused on finding new, safe, and affordable bioactive compounds with anti-mutagenic and anti-cancer properties from plants, vegetables, and fruits to develop functional foods or anti-cancer medications [1,2]. Since cancer remains a leading cause of mortality, studying plant-derived components with anti-cancer activity and lower toxicity has become a vital area of research [3,4,5]. Within this broader search for plant-based therapeutics, sweet potatoes (*Ipomoea batatas*) are well known for their impressive nutritional value and health advantages [3]. It is a globally valuable crop, ranking as the seventh greatest-yielding food crop worldwide and the fifth in tropical regions due to its high nutritional value and ability to adapt to various climates and farming practices [3,6]. In addition to providing essential nutrients, its leaves are rich in polyphenols, which enhance their anti-cancer potential, making it an important crop for future superfoods [3,4,7]. Since sweet potato leaves (SPL) yield crops over several harvest cycles, it can serve as a valuable leafy vegetable in many developing countries, providing protein, fiber, vitamins, minerals, and antioxidants—and showing promise for improving dietary health [7,8].

Building upon these nutritional and phytochemical strengths, several studies have focused on evaluating the role of SPL in disease prevention. SPL are recognized as a promising dietary anti-cancer resource against colon, cervical, breast, prostate, colorectal, and lung cancer cells because of their high phytochemical content [9,10]. Polyphenols from SPL demonstrate in vitro or in vivo physiological activities such as anti-mutagenic, anti-diabetic, radical scavenging, anti-cancer, and anti-bacterial effects [5,11,12]. In addition to these findings, another study indicated that anthocyanins from SPL exhibit significantly stronger effects against colon and cervical cancer cells [13]. Moreover, within the category of protein-based bioactives, the IbACP (*Ipomoea batatas* anti-cancer peptide) can significantly inhibit the proliferation of cancer cells through the induction of DNA damage and fragmentation via the mitochondria-dependent pathway that activates cell death biomarkers, as well as reducing the survival rate of carcinoma cells [5]. Similarly, at the extract level, methanolic extract from SPL has shown anti-mutagenic effects on human stomach cancer cells and rat liver epithelial cells [14]. Furthermore, extending these observations to in vivo models, SPL extract demonstrated a remarkable anti-proliferative effect on prostate cancer in mice during oral administration [15].

To expand the evidence on compound diversity, nine flavonoid components were isolated from SPL and evaluated for their anti-leukemic activity. Among them, cynaroside, nepitrin, and yuanhuanin showed notable potential against acute myeloid leukemia (AML), highlighting their promise as candidates for AML therapy [16]. Similarly, an Okinawan SPL extract has been proven to suppress the formation and growth of colon cancers in mice [17]. Despite these promising findings, for effective cancer prevention and treatment, natural compounds should utilize nano delivery systems, eutectic technologies, and precision nutrition to accelerate their clinical application, providing safer and more personalized strategies [18]. Because of the growing biological importance of sweet potato, research on it has gained significant considerations from scientists, and the quantity of relevant publications has increased rapidly [19]. Usually, all portions of the sweet potato plant (stem, leaf, and root) can be consumed, and these parts can vary in nutrients, non-nutrients, and anti-nutrients [20]. However, compared to the roots of sweet potato plants, less research has been performed on studying the role of SPL in anticancer potential [21]. While numerous studies have highlighted the anticancer potential of SPL and described various mechanisms by which their bioactive compounds act, no systematic comparison or combination of these mechanisms has been conducted yet.

In light of these gaps and emerging findings, this systematic review aims to consolidate and critically analyze the anticancer mechanisms of bioactive compounds in SPL with an emphasis on molecular pathways, compound-specific activities, and extract-dependent variations. This review will provide insights to support future anticancer research and therapeutic development of SPL and highlight directions for future research that may accelerate the development of SPL-based anticancer therapies.

## 2. Materials and Methods

### 2.1. Search Strategy

This systematic review was carried out following the Preferred Reporting Items for Systematic Reviews and Meta-Analyses (PRISMA) guidelines [22]. Scientific articles and review articles written in English and published between 2015 and 2025 were studied in the following databases: PubMed (National Library of Medicine—(https://www.ncbi.nlm.nih.gov/pubmed), Springer (https://www.springer.com), Google Scholar (https://scholar.google.com), Wiley Online Library (https://onlinelibrary.wiley.com), Science Direct (https://www.sciencedirect.com). All of these were accessed on 28 October 2025.

To conduct an efficient search, the keywords were selected carefully to specify more about searching for the SPL anticancer roles rather than other parts of the plants. Some other exclusion and inclusion criteria were also applied. Keywords used included: “SPL,” “*Ipomoea batatas* leaf,” “anticancer,” “cancer,” “apoptosis,” “cell cycle arrest,” and “molecular pathway”. Combine keywords using Boolean operators AND, OR, and quotation marks were also applied, including- “SPL” AND anticancer AND apoptosis, “*Ipomoea batatas*” AND cancer AND “PI3K/Akt”, “sweet potato leaf extract” AND “cell cycle arrest” OR “Bcl-2”. Additionally, combinations of the following keywords were searched: “*Ipomoea batatas* leaves” + “anticancer”, “sweet potato leaf extract” + “apoptosis”, “SPL” + “PI3K/Akt pathway”.

### 2.2. Data Selection

The chosen studies from the listed databases were analyzed in divergent phases. Primarily, the title and abstract from the selected scientific writings were evaluated based on the keywords and the exclusion/inclusion criteria. Next, the duplicates were excluded to avoid repetition. In the further steps, the selected articles were thoroughly read, and based on the selection criteria, exclusion was made for unsuitable ones. Additionally, reference checks were performed for further cross-checking of related articles on the same topics. The inclusion and exclusion criteria for data selection are summarized in Table 1.

### 2.3. Search and Selection Summary

**Figure 1:**
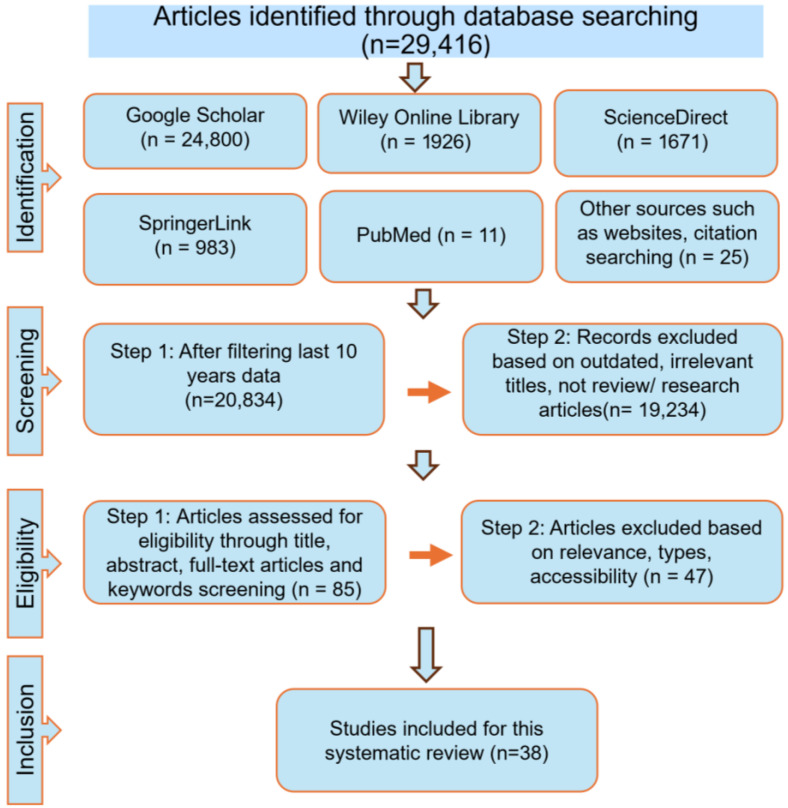
The flowchart illustrates the study selection process.

After searching the PubMed, ScienceDirect, Google Scholar, SpringerLink, and Wiley Online Library databases, a total of 29,416 records were identified. Limiting the search to publications from the past 10 years reduced this number to approximately 20,834. Further refinement to include only research and review articles yielded about 1600 records. Based on titles and keywords, and after applying inclusion and exclusion criteria with the use of Boolean operators, 39 studies are finally selected for this review (Provided in a Supplementary Materials). The database search and selection process followed the PRISMA flowchart, encompassing the stages of identification, screening, eligibility assessment, and final inclusion, as summarized in Figure 1.

## 3. Bioactive Compounds in SPL and Their Anticancer Mechanism

### 3.1. Bioactive Anticancer Compound in SPL

SPL mainly contains diverse bioactive compounds, such as tannins, anthocyanins, polyphenols, sterols, flavonoids, fibers, minerals, and vitamins [8,23]. Additionally, SPL is highly enriched with linoleic and α-linolenic acids [24,25]. In previous research, it was found that SPL had higher total polyphenol content (TPC) and total flavonoid content (TFC) exceeding that of sweet potato root, flesh, peel, and common vegetables [26,27]. The major polyphenols present in SPL include caffeic acid and various caffeoylquinic acid derivatives, such as 4,5-di-O-caffeoylquinic acid, 3,4,5-tri-O-caffeoylquinic acid, 3-mono-O-caffeoylquinic acid, 3,4-di-O-caffeoylquinic acid, and 3,5-di-O-caffeoylquinic acid [28]. Studies also showed that SPL contains a large amount of flavonoids such as anthocyanins, quercetin, myricetin, luteolin, apigenin, etc. [24]. Flavonoids perform as dietary anticarcinogenic agents by advancing the protection of cellular components from peroxidation and oxidative damage [29]. The amount and number of bioactive compounds present in SPL vary significantly among different cultivars, and such variation has significant implications for anticancer studies of SPL extracts. For instance, a study of nine European-grown sweet potato cultivars found that the total polyphenolic content in leaves ranged from 148 mg/100 g dry matter to 14,038.6 mg/100 g, subject to cultivar and harvest stage, the cultivar Carmen Rubin, had the maximum level at the initial stage [24]. Another study comparing three varieties including purple-fleshed “P40”, orange-fleshed “Beauregard” and white-fleshed “Bonita”, the P40 leaves contained 32.7 ± 2.9 mg anthocyanins/kg DW, whereas Beauregard and Bonita had 334 ± 60.9 mg/kg DW and 563 ± 50.4 mg/kg DW, respectively, and TPC (Folin–Ciocalteu) were 36.8 ± 4.8 mg GAE/g DW (P40), 41.2 ± 5.0 mg GAE/g DW (Beauregard) and 46.7 ± 2.1 mg GAE/g DW (Bonita) [30]. A study of 40 cultivars in China found that the variation in leaf total polyphenol content ranged from ~7.39 g to ~14.66 g per 100 g dry weight (DW) across cultivars [8]. Collectively, these studies clearly show that cultivar identity is one of the dominant determinants of SPL bioactive composition.

SPL flavonoid content also varies greatly between cultivars, and leaf color has a significant impact on the content of flavonoids; for instance, green SPL mostly contains apigenin, while purple SPL contains cyanidin, quercetin, myricetin, and luteolin [15,31]. The most common flavonoid group among these substances is anthocyanins, which are present in SPL at concentrations around 2.5 times greater than those in spinach [32]. Additionally, the concentration varies by variety, with purple SPL having a far greater anthocyanin content than leaves that are red, yellow, or green [33]. In addition to flavonoids, SPL are high in carotenoids, especially lutein, which ranges from 34 to 68 mg/100 g across types [34]. SPL also contains other phytochemicals—including alkaloids, anthraquinones, oxalates, and steroids—at concentrations of 345.7, 328.4, 1.66, and 0.375 mg/100 g DW, respectively, while compounds such as phytic acid, cyanide, saponins, and tannins are present in relatively low amounts [35]. These data suggest that the high bioactive content of SPL not only depends on varieties and cultivars but also depends on leaf and flesh colors.

**Figure 2:**
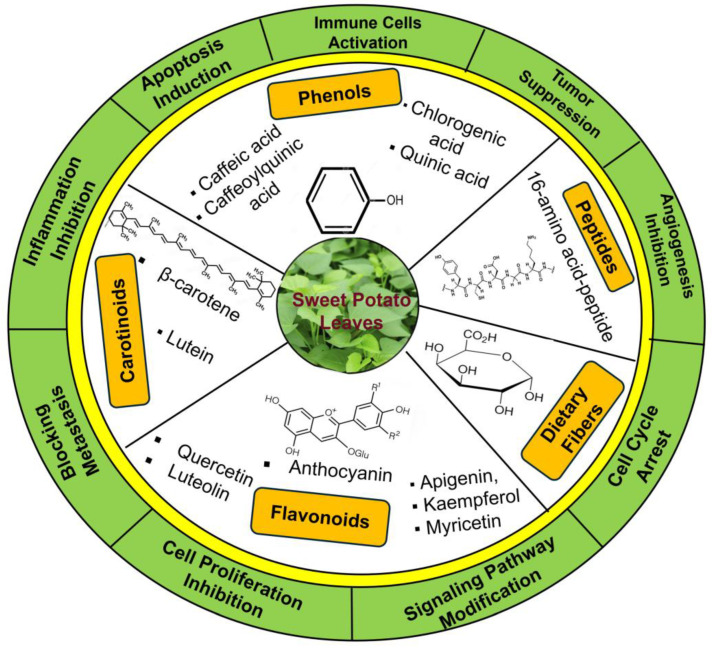
Overview of bioactive compounds present in SPL and their proposed anticancer activities. The figure summarizes key phytochemicals, including phenolics, flavonoids, carotenoids, and anthocyanins, along with their potential anticancer mechanisms.

The bioactive compounds recovered from plant extract also depend on the developmental stages of the leaf and the extraction procedures [26,31]. Investigation including ten different types of extraction processes of SPL showed that ethanolic extraction resulted in the highest flavonoid recovery, whereas acetonic extraction exhibited maximum phenolic recoveries [31]. Several other studies also suggest that the solvent choice strongly determines the extraction efficiency and polyphenolic profile of SPL [31,36]. Thus, extraction methodology introduces another layer of variability that can influence the abundance and composition of bioactive constituents. The leaves also demonstrate anti-proliferative properties by decelerating cancer cell expansion and division. SPL also includes substances that can regulate immune responses, potentially enabling the ability of body to identify and damage cancer cells [7,37]. The SPL has the highest amount of dietary fiber content compared to stems and stalks [29,38]. Additionally, the high dietary fiber content is able to control regular bowel movements and help in the eradication of potential carcinogens [7,39]. The key phytochemicals found in SPL; their potential anticancer functions are summarized in Figure 2.

### 3.2. Anticancer Mechanism of Phenolic Compounds in SPL

Polyphenols, a wide range of plant-based secondary metabolites, occur in varying concentrations and are known to interact with biological tissues, leading to multiple physiological effects [40]. Different types of phenolic acids, such as Caffeic acid derivatives, Caffeoylquinic acid derivatives, Chlorogenic acid, and Quinic acid, found in SPL have shown anticancer and antimutagenic activity [41,42].

#### 3.2.1. Apoptosis Induction

**Figure 3:**
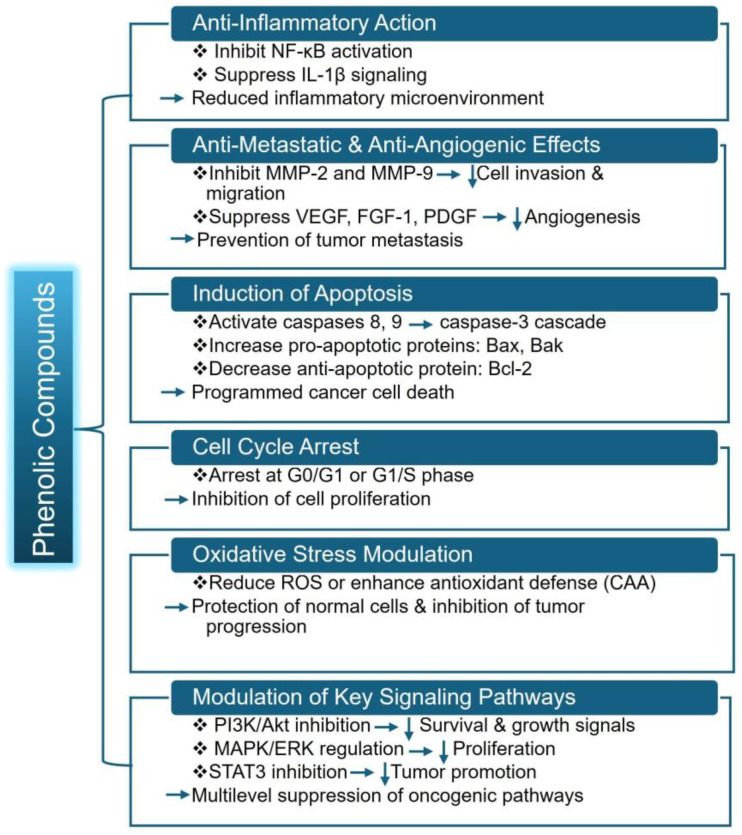
Overall mechanism of phenolic compounds from SPL. Here, NF-κB (Nuclear Factor kappa-light-chain-enhancer of activated B cells), IL-1β (Interleukin-1 beta), MMP-2 (Matrix Metalloproteinase-2), VEGF (Vascular Endothelial Growth Factor), FGF-1 (Fibroblast Growth Factor-1), PDGF (Platelet-Derived Growth Factor), Bax (Bcl-2 Associated X Protein) and Bak (Bcl-2 homologous antagonist/killer protein), ROS (Reactive Oxygen Species), STAT3 (Signal Transducer and Activator of Transcription-3). Downward arrows indicate inhibition or reduction.

SPL polyphenol extracts and isolated caffeoylquinic/chlorogenic derivatives trigger programmed cell death in prostate cancer cells by enhancing caspase-3 activity [15]. This same study also showed that SPL extract affects apoptosis-related molecules by Bcl2 (B cell lymphoma/leukemia-2 protein) inhibition, BAX (Bcl-2 associated X protein) enhancement, cytochrome c liberation, and initiation of downstream apoptosis pathways. The tumor-suppressive effects of caffeic and chlorogenic acids are also recently supported by a study where these compounds, along with phenolic acid, exposed C32 cells to a static magnetic field to trigger apoptosis and also regulated the expression of genes involved in apoptosis-controlling proteins [43]. Together, these findings illustrate that SPL phenolics can promote cell death through both mitochondrial and gene-regulatory mechanisms. The overall mechanism of phenolic compounds from SPL is represented in Figure 3.

In addition to prostate and melanoma models, the study examined the apoptosis-promoting and angiogenesis-inhibiting activities of eight bioactive constituents isolated from the vines of sweet potatoes*,* comprising chlorogenic acid, various dicaffeoylquinic acids (3,4-, 3,5-, 4,5-), 1,3,5-tricaffeoylquinic acid, N-trans-feruloyltyramine, and the coumarins esculetin and scopoletin [44]. Among them, 1,3,5-tricaffeoylquinic acid revealed the strongest cytotoxicity in A2780 ovarian cancer cells by triggering both intrinsic and extrinsic apoptosis. It activated caspase-8 and caspase-9, giving rise to caspase-3 and PARP (Poly ADP-ribose Polymerase) cleavage [44,45,46]. This dual pathway activation highlights that 1,3,5-tricaffeoylquinic acid is potentially an anticancer agent against ovarian cancer [44]. Not only from SPL, but phenolic acids from many other sources also show potential anticancer effects, such as chlorogenic acid, which inhibited breast cancer cell growth by reducing viability, proliferation, migration, and invasion while inducing apoptosis [47].

#### 3.2.2. Modulation of Oncogenic Signaling Pathways

Phenolic compounds derived from plants inhibit cancer cells’ initiation and proliferation by gene modulation in different processes, for example, controlling normal cells transforming into oncogenic ones, modulating tumor cell proliferation and development, controlling angiogenesis, and the metastasis process [48]. Inhibition of human colon cancer DLD-1 development, leukemia HL-60 cells, and stomach Kato III cancer cells is greatly induced by caffeoylquinic acid [11,41]. In addition to these cytotoxic effects, enhancing the activity and expression of caspase-3 activity of c-Jun, which acts as an apoptosis-related gene, is also achieved by Caffeoylquinic acid derivatives [11,41]. One of the research groups also reported that SPL extracts inhibit lipopolysaccharide-induced inflammation in RAW 264.7 murine macrophage cells by suppressing NF-κB and IL-1β signaling pathways, as well as reducing IKK-α (IκB Kinase alpha) and IκB-α (inhibitor of NF-κB alpha) phosphorylation and iNOS (inducible nitric oxide synthase) expression [49]. Similarly, A recent study demonstrated that purified phenols from SPL extract showed mediating anti-inflammatory activity through suppression of NF-κB signaling, including inhibition of IκBα phosphorylation/degradation and restriction of p65 nuclear translocation, while also suppressing MAPK (Mitogen-Activated Protein Kinase) signaling by reducing phosphorylation of p38, ERK (Extracellular signal-Regulated Kinase), and JNK (c-Jun N-terminal Kinase) [50]. These types of anti-inflammatory mechanisms can support anticancer functions by disrupting the inflammatory pathways that promote tumor growth [51,52]. A summary of studies on the anticancer potential of SPL is presented in Table 2.

#### 3.2.3. Inhibition of Metastasis and Oxidative Stress Modulation

Several phenolics interfere with metastasis-related processes by down-regulating metastasis-associated enzymes and pathways, thereby reducing invasive behavior in various cancer models. Chlorogenic acid inhibited NF-κB and EMT signaling, suppressed tumor progression, and increased lifespan in a 4T1 mouse model. It normally suppresses lung metastasis of 4T1 cells by increasing CD4+ and CD8+ T cells of the mice spleen, which enhances the antitumor immunity [41]. Inhibition of metastasis also occurs with quercetin by suppression of alterations in the extracellular matrix and lowering tumor progression and development through suppressor matrix metalloproteinase [54]. It also boosts the proapoptotic proteins expression and suppresses Bcl-2 expression [54,55]. Phenol from plants induces cancer cell death by downregulating the PI3K and Akt pathway, reducing cell proliferation by targeting the cyclin-dependent pathway, and activating some factors, such as transcription factors NF-kβ, NRF2, and STATs. It also downregulates some other factors, such as angiogenic factors and histone deacetylase [48]. Polyphenols from SPL decreased VEGF-driven cell proliferation, motility, and capillary-like structure formation in human umbilical vein endothelial cells (HUVECs) [56,57]. These polyphenols (e.g., caffeoylquinic acids) can also downregulate MMP-2 and MMP-9, limiting tumor cell proliferation and invasion. They also inhibit pro-angiogenic factors such as VEGF, FGF-1, and PDGF, which limit new blood vessel creation and hence prevent metastatic propagation [41,44,56]. The immune cells and tumor cells contribute differently to metastasis inhibition. For instance, the natural killer (NK) cells can eliminate metastatic cancer cells without prior activation by antigen-presenting cells [58]. Thus, NK cell immune regulations are controlled through a balance between activating and inhibitory receptor signaling. On the other hand, metastatic tumor cells employ multiple mechanisms to evade immune attack [58]. The differences between immune cells and tumors in metastasis inhibition are one of the key regulatory components of anticancer mechanisms. SPL polyphenols can also act on cancer cells via oxidative stress modulation—especially caffeoylquinic acids—significantly reduce intracellular ROS and boost cellular antioxidant defenses, protecting normal cells from oxidative damage while helping to inhibit tumor progression [59].

#### 3.2.4. Enzyme Regulation, Cell Proliferation Inhibition, and Cell-Cycle Arrest

Building on their effects on metastasis and oxidative stress, phenolic compounds from SPL can inhibit tumor metastasis-related enzymes, reduce tumor cell proliferation, and arrest cell-cycle progression (G0/G1 or S phase) that can contribute to decreased tumor growth in vitro and in vivo [3,11,41]. Chlorogenic acid is reported to act as a potential inhibitor of enzymes that have roles in tumor metastasis in human lung cancer cells [41,60]. In human lung cancer cells, Chlorogenic acid has shown strong and specific inhibition of matrix metalloproteinase-2 and matrix metalloproteinase-9, which act as angiogenic enzymes that have a key responsibility in tumor invasion and metastasis [41,56]. A type of polyphenol named anthocyanins comprises different pigments in sweet potato and other plants, and they directly react with enzymatically activated carcinogens and inhibit mutagenesis [14]. Research investigating lipid-soluble polyphenols from sweet potato demonstrated that they induce cell cycle arrest at the G0/G1 phase by inhibiting the Akt activity and thus increase the effectiveness of tumor-suppressing agents [61].

### 3.3. Anticancer Mechanism of Flavonoid Derivatives in SPL

**Figure 4:**
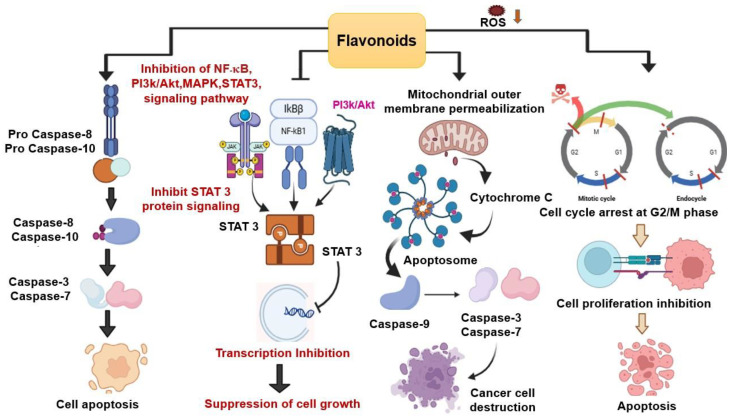
Overall Anticancer Mechanism of Flavonoids. Flavonoids induce arrest of cells in the G2/M phase of the cell cycle and initiate ROS-mediated apoptosis by inhibiting cell growth. cell growth. The downregulation is denoted by the orange arrow. The red lines denote the check points for mitotic cycle arrest while dashed red line represent skipping of M phase. By blocking important signaling pathways, such as NF-κB, MAPK, and STAT, flavonoids cause the down regulation of several mediators and limit cell growth. P represents phosphorylation which is a process for activating or deactivating pathways. Flavonoids also induce mitochondrial apoptosis by activating specific signaling pathways, such as the cytochrome c translocation into the cytoplasm, which promotes the formation of apoptosomes. When apoptosomes form, caspase 9 is activated, followed by caspase 3 and 7, which trigger the cellular components to undergo apoptosis. Another flavonoid-mediated apoptotic pathway is depicted in the image, which involves the activation of Caspase 8 and 10, followed by the production of Caspase 3 and 7, leading to the destruction of cancer cells.

Different types of flavonoids exhibited their function in cancer metastasis and apoptosis. Flavonoids such as, Anthocyanins, Apigenin, Kaempferol, Myricetin, Fisetin, Morin, and Luteolin exhibit anticancer roles in vitro and in vivo [41,53]. Quercetin from SPL inhibits the viability of various cancer cell lines, including human breast cancer, leukemia, colon, and ovarian carcinoma cells, while other flavonols such as, yuanhuanin, nepitrin, and cynaroside also show potential anti-leukemia activity by limiting AML cell proliferation [16,54]. Overall, the anticancer mechanism of flavonoids is represented in Figure 4.

#### 3.3.1. Cell-Cycle Regulation and Antioxidant Activities

In addition to their general anticancer roles, flavonoids of SPL contribute significantly to cell cycle control and antioxidant defense, which makes it a potential candidate for chemopreventive action. Several studies have shown that SPL-derived flavonoids can induce cell cycle arrest at different checkpoints, particularly the G2/M phase. In the case of breast cancer, Isoflavone genistein initiated induction of G2/M phase cell cycle arrest and performed subsequent ROS-dependent apoptosis [56]. Another flavonoid named Daidzein stimulated apoptotic cell death in MCF-7 breast cancer cells through ROS generation [57]. By enhancing the ROS production and activation of the mitochondrial apoptotic pathway, Flavanone hesperetin induced promotion of programmed cell death in esophageal cancer, hepatocellular carcinoma, and human breast carcinoma (MCF-7) cells [62]. Anthocyanins can also inhibit cell growth by altering regulatory proteins such as cyclin A, cyclin D1, p21, p27, and p53 [13]. Flavonoids perform a great part in the apoptosis pathway by having dual action in the ROS pathway- first, they act as antioxidants, secondly, they perform the action of prooxidants that trigger the apoptosis [16,62]. Together, the ability of SPL flavonoids to modulate oxidative stress and regulate cell cycle progression highlights their potential as natural chemopreventive agents.

#### 3.3.2. Inhibit Proliferation

Flavonoids in SPL have been verified to suppress cancer cell growth and proliferation in multiple cell types. This anti-proliferative effect is mediated by pro-oxidant activity of flavonoids through blockade of EGFR/MAPK, PI3K, and Akt activity [54,62]. Flavonoids exert anticancer effects in lung cancer by inhibiting tyrosine kinase receptors and blocking MAPK, PI3K/Akt, and JAK/STAT pathways, thereby inactivating STAT-3, suppressing transcriptional activity, and reducing cancer cell proliferation [63]. A type of anthocyanin named Cyanidin-3-glucoside decreased lung tumor growth by acting as an efficient mediator in the ApcMin intestinal cancer model, resulting in exhibited diminished metastatic progression in the A549 nude mouse xenograft system [41]. In another study, the potential mechanisms of Cyanidin-3-glucoside in lung adenocarcinoma were presented, showing that it exerts anticancer effects through suppression of TP53I3 (Tumor Protein P53 inducible Protein 3) expression and blockade of the PI3K/AKT/mTOR signaling cascade [64]. Similarly, quercetin inhibits breast cancer stem cells by reducing the expression of chemokine receptor type 4 (CXCR4), mucin 1 (MUC1), aldehyde dehydrogenase 1A1 (ALDH1A1), and epithelial cell adhesion molecule (EpCAM) both in vitro and in vivo, while also preventing tumor metastasis in the CD44^+^/CD24^−^ population and downregulating estrogen receptor α and PI3K/Akt/mTOR signaling pathways [65].

#### 3.3.3. Apoptosis Induction

There is a close relationship between the anticancer properties and the anthocyanin, as the anthocyanin synthase gene correlated to the anticancer mechanism [54]. The anthocyanins induce apoptosis by both the extrinsic and intrinsic pathways [13,66]. Cytochrome C is circulated during the intrinsic pathway as the anthocyanin enhances mitochondrial potential [13,67]. Additionally, the caspase-dependent pathway and cancer cell death are upregulated by applying anthocyanin. The FAS and FAS ligand expressions are also regulated by anthocyanin in the case of the extrinsic pathway [13]. Furthermore, mitogen-activated protein kinase pathway activation is arrested by anthocyanins, which inhibit the process of carcinogenesis [13,66]. Flavanone naringenin showed anti-cancer impacts on choriocarcinoma JAR and JEG 3 cell lines, initiating programmed cell-death signaling in human epidermoid carcinoma A431 cells [62,68]. A recent study based on network pharmacology and bioinformatics analysis of flavonoid components of SPL revealed that they can induce apoptosis in AML cells [16]. Extracts from SPL have shown strong antileukemic activity, with metabolomics and pharmacology analyses identifying cynaroside, nepitrin, and yuanhuanin as key flavonoids targeting CASP3, KDR, EGFR, and SRC in AML. Their effects were validated through both cell-based and animal studies, highlighting their potential as promising treatments for AML [16]. A related study showed that quercetin enhances the anticancer effects of bromodomain and extra-terminal domain (BET) proteins in pancreatic cancer by promoting apoptosis, reducing proliferation, and suppressing sphere formation in vitro and in vivo, partly through hnRNPA1 downregulation and decreased anti-apoptotic protein Survivin expression, while its combination with BET inhibitors further improves tumor-growth suppression [69].

#### 3.3.4. Modulation of Signaling Pathways

Beyond apoptosis and proliferation inhibition, anthocyanins have roles in important signaling pathways, such as AMPK, PI3K/AKT/mTOR, and JAK-STAT. Among them, the JAK-STAT pathway—specifically the inhibition of STAT3- is extremely associated with tumor development [70]. For instance, anthocyanins named peonidin-3-glucoside and cyanidin-3-glucoside can block HER-2 phosphorylation, leading to decreased cancer cell migration and invasion [71]. Another flavonoid, luteolin, suppressed proliferation and induced dose-dependent apoptosis in MDA-MB-231 breast cancer cells while reducing telomerase levels by inhibiting IκBα phosphorylation and downstream c-Myc, thereby suppressing hTERT expression [72].

### 3.4. Bioactive Peptides in SPL

Anti-cancer peptides, which are highly specific and effective, are already approved and undergoing clinical trials for cancer treatment [73,74]. Peptides from different plants have exhibited their biological potential in treating cancer, for example, hindering the proliferation of cancer cells and reducing pathogen attacks [5]. These are also beneficial due to their easy absorption rate and convenient route of administration [5,75]. SPL contains peptide sequences having anti-cancer roles. The 16-residue peptide termed IbACP has shown anticancer activity in treating pancreatic cancer [5]. Overall, the bioactive peptides have gained great focus in the field of cancer treatment due to their beneficial roles, such as higher selectivity, lowered toxicity, and increased adaptability in targeting the specific molecular pathway correlated to cancer [74].

#### 3.4.1. Cancer Cell Proliferation Inhibition

**Figure 5:**
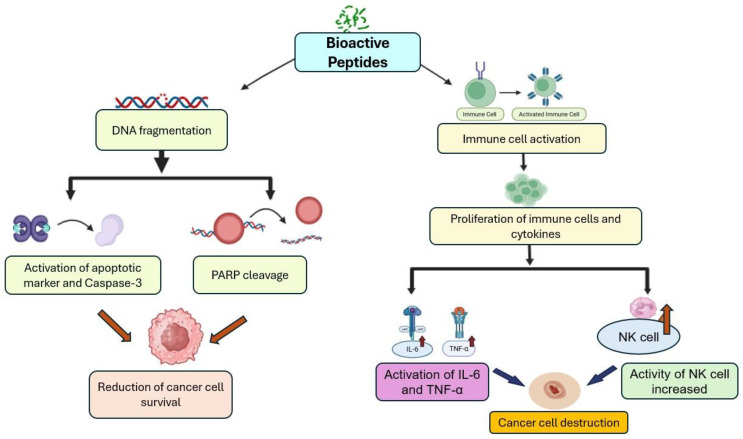
An overview of anticancer mechanisms exerted by bioactive peptides of SPL.

IbACP-dependent Cancer cell proliferation inhibition is performed by DNA fragmentation induction, resulting in activation of apoptosis markers and reduction in the cancer cell survival rate [5]. Panc-1 cells proliferation is inhibited by IbACP, resulting in cellular elicits apoptosis in a dose-responsive way, and also activating caspase-3 and poly (ADP-ribose) polymerase [5]. Several bioactive peptides from other plants, such as amaranth, soybean, seaweed, and *Raja porosa*, have also shown antiproliferative and anticancer activities [76,77,78,79]. Lectins derived from the seaweed *Eucheuma serra* exert anticancer effects through cytotoxicity, initiating apoptosis, and restricting tumor development [77,80]. Additionally, the FIMGPY peptide from *Raja porosa* demonstrates significant antiproliferative activity [76]. The overall anticancer pathway of the bioactive peptide of SPL is summarized in Figure 5.

#### 3.4.2. Immune Cell Activation

Bioactive peptides may trigger the activation or the proliferation of various immune cells and also cytokines that perform a crucial role in defending against cancer cells and pathogens [74]. Additionally, they can inhibit the cell migration process through various mechanisms, such as tissue remodeling, chemotaxis, and cell adhesion, which are particularly important to stop cancer metastasis [74,81]. In addition to SPL, peptides from different plants have also shown anticancer properties. For instance, five peptides from beans showed anticancer properties on human colon cancer cell lines through cell cycle arrest and controlling cell proliferation [81]. Multiple wheat-derived peptides have been demonstrated to possess immunomodulatory effects by stimulating phagocytosis, promoting lymphocyte proliferation, enhancing the production of nitric oxide, IL-6, and TNF-α, increasing NK cell activity, and diminishing the expression of cytokines involved in inflammation, including Th1 and Th17 [82,83].

### 3.5. Dietary Fiber Anticancer Mechanism

Dietary fibers also have a great impact in fighting against cancer [41,84]. Research has shown that enhanced consumption can be helpful in protecting against forms of cancer, such as breast cancer and rectal cancer [85,86]. A different study indicated that the intake of most dietary fiber reduced the 13% mortality rate in cancer compared to those who consumed the least [85,87]. However, excess intake of dietary fiber causes some adverse effects, such as abdominal cramps, flatulence, diarrhea [85,87]

In the case of colon cancer etiology, dietary fiber can bind with bile acids, altering the enterohepatic axis, resulting in a reduction in the cholesterol level [85,88]. Dietary fiber can remove stomach nitrite, reducing nitroso compounds under an acidic environment, which is responsible for gastric cancer [85,89]. To reduce breast cancer risk, dietary fiber inhibits β-glucosidase activity of bacteria and decreases the concentration of estrogen, resulting in inhibition of estrogen reabsorption in the colon and enhancement of excretion of fecal estrogen, which results in a reduction in breast cancer risk [85,90,91].

### 3.6. Carotenoids Anticancer Mechanism

Carotenoids present in the leaves also have notable anticarcinogenic activities [41,84]. For instance, inflammation that is responsible for breast cancer can be controlled by carotenoids [92,93]. In lung cancer, carotenoids can protect the lungs at earlier carcinogenic phases by their antioxidant activity [94,95]. Cell culture studies have reported that a certain amount of beta carotene is able to reduce the expression of anti-apoptotic proteins, which include Bcl-2 and PARP [96]. Additionally, lycopene can modulate proliferation and enhance apoptosis in MCF-7 cells.

## 4. Enhancing Conventional Cancer Therapies with Bioactive Compounds from SPL

Conventional cancer treatments such as radiotherapy, chemotherapy, and anticancer drugs often lose effectiveness due to multidrug resistance (MDR) and the inability of some therapies to adequately reach malignant tissues [97]. Plant-derived bioactive compounds have therefore emerged as promising adjuncts capable of overcoming these limitations. Recent research shows that bioactive constituents of SPL can be combined with standard chemotherapeutics to enhance efficacy and reduce systemic toxicity by exerting potent antioxidants, antiproliferative, and anti-inflammatory activities that modulate oxidative stress, apoptosis, and drug sensitivity in cancer cells [58,59].

Several oncogenic pathways often show chemoresistance in some cancer treatment cases, motivating research into combining SPL-derived compounds with conventional therapies to achieve stronger anticancer effects [97,98]. For example, colon cancer often develops resistance to ionizing radiation (IR), necessitating higher doses that risk damaging healthy tissues. However, Li et al. demonstrated that quercetin markedly enhances radiosensitivity by inhibiting proliferation, promoting apoptosis, reducing angiogenesis and metastasis, and suppressing Notch-1 signaling in HT-29 and DLD-1 cells—thereby increasing responsiveness to radiotherapy [98]. Some other studies also showed that quercetin, combined with either cisplatin or doxorubicin, enhances anticancer effects by promoting apoptosis and antiproliferation in cell lines and inducing complete tumor regression with strong immune responses in mice [99,100]. Other SPL-associated flavonoids, such as resveratrol (commonly found in purple sweet potato), also demonstrate synergy with chemotherapeutics. Chung et al. showed that resveratrol combined with 5-fluorouracil (5-FU) reduces STAT3 phosphorylation and binds to the hTERT promoter, inducing apoptosis and resensitizing colon cancer cells to chemotherapy [101]. In line with this, Kweon et al. reported that resveratrol reverses doxorubicin resistance in AML by suppressing MRP1, increasing intracellular drug retention, and restoring chemosensitivity [102]. Furthermore, SPL polyphenols and carotenoids may enhance intracellular drug accumulation, lower toxicity, and downregulate anti-apoptotic proteins and MDR transporters, improving responsiveness to paclitaxel, cisplatin, and doxorubicin [61,70,74].

Luteolin, another flavonoid found in SPL, is well recognized for its chemopreventive and chemosensitizing roles. In addition, specific evidence of sweet potato–derived luteolin in combination therapy is limited; several studies highlight the compound’s synergistic potential. Jeon et al. demonstrated that luteolin combined with celecoxib synergistically suppressed tumor growth in multiple breast cancer cell lines, mediated through Akt inhibition and cell-line–specific modulation of ERK signaling [103]. Likewise, Ren et al. showed that luteolin enhances the antitumor activity of oxaliplatin in gastric cancer by promoting cytochrome-c release and activating cleaved caspase-3 and BA [104]. Beyond direct anticancer effects, the antioxidant properties of SPL bioactives may also protect healthy tissues during chemotherapy by reducing oxidative damage, improving patient tolerance, and lowering adverse effects [59]. Collectively, these interactions highlight the promise of SPL extracts as adjuvant agents capable of reducing drug dosage without compromising therapeutic outcomes.

### Industrial Scalability, Applicability, and Sustainability

SPL bioactive compounds hold substantial industrial scalability potential due to the crop’s agronomic adaptability, high biomass yield, and cost-effective cultivation across multiple climatic zones [19]. Recent advances in extraction techniques, including microwave-assisted, supercritical CO_2_, and green solvent-based methods, have significantly improved the yield, purity, and stability of these phytochemicals, facilitating their integration into industrial supply chains while minimizing environmental impact [40,105]. Large-scale extraction process using NKA-II macroporous resin achieved high purity and identified 19 major compounds in SPL, supporting the utilization of this agricultural by-product for functional foods, dietary supplements, or fortified beverages as natural anti-inflammatory sources [50].

From an application perspective, sweet potato bioactives show significant potential in the development of pharmaceutical, nutraceutical, and functional food products. Their integration into food matrices meets the growing consumer demand for clean-label and health-promoting ingredients, including health beverages, fortified snacks, and natural colorants [8,20]. For instance, purple-fleshed SPL contain natural purple, burgundy, pink, and green pigments that can replace synthetic colorants in various food and beverage applications [106]. Furthermore, bioactive compounds such as carotenoids, flavonols, and dietary fiber can be incorporated into fortified foods. SPL provides potent bioactive compounds with strong antioxidant, nutritional, cosmetic, and therapeutic potential, enabling their use in functional foods, nutraceuticals, cosmetics, and value-added products that support circular agriculture and improved health [7,106].

## 5. Limitations and Scope

Despite having numerous promising mechanisms, several limitations constrain the translation of SPL bioactives into clinical use. Most studies summarized in Table 2 rely heavily on in vitro cancer cell models and a limited number of in vivo animal experiments, which fail to capture the complexity of human tumors, including microenvironmental interactions, immune modulation, and metastatic behavior. As a result, current findings remain preliminary and cannot yet be extrapolated to human therapeutic outcomes [107]. Moreover, the observed cytotoxic, antiproliferative, and pro-apoptotic effects of SPL compounds are largely correlative, with limited mechanistic validation across diverse cancer types. The molecular mechanisms—such as activation of apoptosis or modulation of PI3K/Akt and MAPK pathways—require further target-specific validation using pathway inhibitors, genetic knockdowns, or reporter assays [107]. Another major limitation lies in the metabolic and pharmacokinetic behavior of SPL bioactives in vivo. Many polyphenolic compounds demonstrate low oral bioavailability and rapid metabolic transformation, undergoing extensive intestinal, hepatic, and microbial metabolism that drastically reduces their systemic concentrations relative to in vitro levels [108,109]. Similarly, anthocyanins—a major class of SPL bioactives—are known for poor absorption, rapid degradation, and extensive metabolism in vivo [110]. Without addressing these pharmacokinetic constraints, the clinical value of SPL compounds remains uncertain.

These limitations highlight several key research gaps: insufficient research on target molecule and anticancer mechanism, the absence of clinical trials or human studies; insufficient understanding of dose–response relationships; lack of standardized extraction and purification methods; limited assessment of synergistic effects with existing chemotherapeutics; and no established strategies to enhance bioavailability. Future research should prioritize advanced in vivo models, targeted delivery systems, standardized extraction protocols, and pharmacokinetic optimization to evaluate SPL’s realistic potential in cancer therapy. The overall gap of research in this area is summarized in Table 3.

## 6. Conclusions

This systematic review explores the anticancer potential of bioactive compounds found in the leaves of sweet potato. Overall, phenolic compounds induce apoptosis, inhibit metastasis, and modulate oxidative stress and oncogenic signaling pathways whereas flavonoids regulate cell cycle progression, trigger ROS-mediated apoptosis, and suppress tumor proliferation via MAPK, PI3K/Akt, and STAT3 inhibition. On the other hand, bioactive peptides perform mainly by inhibition of cancer cell proliferation, inducing DNA fragmentation and caspase activation, and enhancing immune-mediated tumor suppression. Carotenoids provide antioxidant protection, reduce anti-apoptotic protein expression, and complement phenolics and flavonoids in promoting apoptosis. Together, these compounds act synergistically to suppress tumor growth, prevent metastasis, and enhance anticancer efficacy, highlighting SPL as a promising multifunctional dietary source for cancer prevention and therapy. These findings suggest that SPL and their bioactive constituents function as potent natural modulators of cancer-related cellular processes. In addition, sweet potato tubers have been extensively studied for their nutritional and therapeutic benefits, the leaves of this plant remain underexplored, which this review aims to address.

This review successfully fulfills its objectives by identifying major bioactive constituents of SPL, summarizing their mechanisms of action, and critically analyzing experimental data supporting their efficacy. In addition, more research is needed; SPL has strong potential to play an important role in anticancer strategies. Incorporating SPL into food systems could enhance nutritional value, support health promotion, and contribute to food sustainability by utilizing agricultural by-products. Overall, SPL holds exceptional potential as a cost-effective, sustainable source of bioactive compounds for cancer prevention and functional food innovation—bridging the gap between nutrition and therapeutic application. By advancing this single agent from in vitro studies to clinically relevant combination strategies, SPL bioactive compounds could become a valuable adjunct in cancer research.

## Figures and Tables

**Table 1:** Criteria for study eligibility.

Criteria	Inclusion Criteria	Exclusion Criteria
Study Type	Peer-reviewed original research articles, literature, and systematic reviews.	Non-peer-reviewed sources, editorials, conference abstracts, book chapters, or blogs.
Time Frame	Published within the last 10 years (2015–2025).	Published before 2015 unless highly relevant and widely cited.
Language	English	Non-English articles without an English translation.
Full Text	Studies with full text available (free or via institutional access).	Studies where only abstracts are available, or the full text is not accessible.
Relevance	Articles that specifically investigate anticancer or antiproliferative effects of SPL.	Studies focusing on other parts of sweet potato (e.g., root, stem) or general dietary/nutritional benefits only.
Subjects/Models	Studies involving in vitro, in vivo, or human clinical trials related to SPL and cancer.	Studies on SPL but not related to cancer, or studies using unrelated models (e.g., soil studies).
Mechanism Studied	Studies investigating bioactive compounds (e.g., polyphenols, flavonoids), apoptosis, and caspase activation.	Studies lacking data on mechanisms or without relevance to cancer pathway modulation.
Plant Parts	Leaves only	Other parts, except leaves, such as the potato, stem, vines, and peels.

**Table 2:** Summary of studies of the anticancer potential of SPL.

Type of Extract	Bioactive Compounds	Type of Cancer Evaluated	IC_50_	Results	Reference
Methanolic Extract	1,3,5-Tricaffeoylquinic Acid	A2780 human ovarian cancer cells	47.43 ± 2.43 μM	Apoptotic death above 37% cells and reduced sustainability to below 25%.	[44]
Methanolic Extract	Flavonoids	acute myeloid leukemia cell line	31.77 μM (HL60 cells) and 18.46 μM (Thp1 cells)	Notable inhibition of AML cell growth.	[16]
Aqueous/ethanolic	Polyphenols, anthocyanins	BT-549 (breast cancer), A549 (lung cancer) cell lines	0.002 µg/µL (BT549 cancer cell line) and 0.0014 µg/mL (A549 cancer cell line)	Apoptosis; anti-proliferative effects.	[21]
Methanol, Ethanol, TFA, Water Extract	Anthocyanins	MCF-7 (breast cancer cells), cervical cancer cells, HeLa, and HCT-116 (colon cancer cell lines)	No IC_50_ value is reported	The highest anticancer activity through apoptosis was observed against HeLa cells.	[13]
Boiling water-extracted PSPL	Flavonoids	Differentiated 3T3-L1 cells	No IC_50_ value is reported	Apoptosis, increase expression of caspase pathway, downregulate inflammation-associated genes.	[53]
16-amino-acid peptide	Peptides	Antiproliferative assay: pancreatic cancer	Not reported	Genomic DNA fragmentation and apoptosis of cancer cells by activation of caspase-3 and poly (ADP-ribose) polymerase.	[5]
Methanolic	Anthocyanins and Polyphenolic Compounds	Antiproliferative assay: Human prostate cancer cell lines	145 µg/mL (prostate cancer C4-2 cells)	Genomic DNA fragmentation and apoptosis of cancer cells. The highest anticancer potency was observed against C4-2 cells.	[15]
Methanolic	Polyphenols	Colon cancer cell, Lung cancer cell, Stomach cancer cell, and Uterus cancer cell	244 µg/mL (human stomach cancer cells), 2125 µg/mL (liver cancer cell), 2495 µg/mL (lung cancer cell)	The strongest anticancer effect was found against stomach cancer cells.	[14]

**Table 3:** An overview of the research gap and suggested future directions.

Research Gaps	Significance	Suggested Future Direction	Evidences
Limited mechanistic insight into molecular targets	The majority of research describes growth inhibition or apoptosis without verifying the precise biochemical mechanisms involved.	To find direct molecular targets (such as PI3K/Akt and MAPK), leverage transcriptomics, proteomics, and gene-silencing studies.	[13,16,70]
Variability across cultivars and growth conditions	Variation makes reproducibility challenging and may cause inconsistent biological result	Perform broad, systematic screening across various cultivars and growth condition	[12,21,29,111]
Lack of human clinical validation	Existing research is limited to in vitro or in vivo models; translational validation for consumption by humans is lacking.	Conduct controlled human trials to determine the pharmacokinetics, safety, and therapeutic effects of SPL bioactive.	[13,16,54]
Inconsistent phytochemical profiling and extraction protocols	Differences in cultivar, solvent, and extraction procedure lead to variations in composition and biological outcomes.	Establish standardized extraction and analytical procedures (HPLC/LC-MS) to ensure consistent characterization.	[44]
Lack of combinatory or synergistic studies	The adjuvant potential of SPL compounds with chemotherapeutics is unknown.	Analyze the synergistic effects of SPL substances with current medications utilizing the combination index or isobologram analysis.	[112,113]

## Data Availability

The data presented in this study are available on request from the corresponding author.

## Supplementary Materials

The following supporting information can be downloaded at: https://www.mdpi.com/article/10.3390/foods15010093/s1, Based on titles and keywords, and after applying inclusion and exclusion criteria with the use of Boolean operators, 39 studies are finally selected for this review [2,3,5,6,7,8,9,10,11,12,13,14,15,16,17,18,19,20,21,24,27,29,34,37,38,39,40,41,42,44,49,50,54,61,70,73,89,114,115].

## Abbreviations

The following abbreviations are used in this manuscript:

AML	Acute Myeloid Leukemia
Akt	Protein Kinase B
BAX	Bcl-2 Associated X Protein
Bcl-2	B Cell Lymphoma/Leukemia-2 Protein
DW	Dry Weight
ECM	Extracellular Matrix
EGFR	Epidermal Growth Factor Receptor
EMT	Epithelial–Mesenchymal Transition
ERK	Extracellular Signal-Regulated Kinase
FGF-1	Fibroblast Growth Factor 1
IbACP	*Ipomoea batatas* Anti-Cancer Peptide
IKK-α	IκB Kinase Alpha
IL-1β	Interleukin-1 Beta
iNOS	Inducible Nitric Oxide Synthase
IκB-α	Inhibitor of NF-κB Alpha
JNK	c-Jun N-Terminal Kinase
MAPK	Mitogen-Activated Protein Kinase
MMP-2	Matrix Metalloproteinase-2
NF-κB	Nuclear Factor Kappa-Light-Chain-Enhancer of Activated B Cells
NO	Nitric Oxide
NRF2	Nuclear Factor Erythroid 2-Related Factor
PARP	Poly ADP-Ribose Polymerase
PDGF	Platelet-Derived Growth Factor
PI3K	Phosphatidylinositide 3-Kinase
ROS	Reactive Oxygen Species
SPL	Sweet Potato Leaves
STAT-3	Signal Transducer and Activator of Transcription 3
TFC	Total Flavonoid Content
TPC	Total Polyphenol Content
VEGF	Vascular Endothelial Growth Factor
