# Supplementary material for: Anticancer Mechanisms of Bioactive Compounds from Sweet Potato (Ipomoea batatas L.) Leaves: A Systematic Review
Source: Foods. 2025 Dec 29;15(1):93. doi: 10.3390/foods15010093 (PMC12785916; doi:10.3390/foods15010093)
Supplement: Supplementary file 1 [file foods-15-00093-s001.zip › foods-3985875-supplementary.pdf]

1. Phahlane, C.J.; Laurie, S.M.; Shoko, T.; Manhivi, V.E.; Sivakumar, D. An Evaluation of Phenolic Compounds, Carotenoids, and Antioxidant Properties in Leaves of South African Cultivars, Peruvian 199062.1 and USA's Beauregard. *Front. Nutr.* **2021**, *8*, 773550. https://doi.org/10.3389/fnut.2021.773550
2. Kato, K.; Nagane, M.; Aihara, N.; Kamiie, J.; Miyanabe, M.; Hiraki, S.; Luo, X.; Nakanishi, I.; Shoji, Y.; Matsumoto, K.I.; Yamashita, T. Lipid-soluble polyphenols from sweet potato exert antitumor activity and enhance chemosensitivity in breast cancer. J. Clin. Biochem. Nutr. 2021, 68, 193–200. https://doi.org/10.3164/jcbn.20-73
3. Silva-Correa, C.R.; Hilario-Vargas, J.; Villarreal-La Torre, V.E.; Calderón-Peña, A.A.; González-Siccha, A.D.; Aspajo-Villalaz, C.L.; Cruzado-Razco, J.L. Potential anticancer activity of bioactive compounds from Ipomoea batatas. Pharmacogn. J. 2022, 14, 650–659. https://doi.org/10.5530/pj.2022.14.84
4. Huang, D.-J.; Lin, C.-D.; Chen, H.-J.; Lin, Y.-H. Antioxidant and antiproliferative activities of sweet potato (Ipomoea batatas [L.] Lam 'Tainong 57') constituents. Bot. Bull. Acad. Sin. 2004, 45, 179–186.
5. Zhang, H.; Zhang, L.; Gao, F.; Yang, S.; Deng, Q.; Shi, K.; Li, S. Purification, composition, and anti-inflammatory activity of polyphenols from sweet potato stems and leaves. Foods 2025, 14, 2903. https://doi.org/10.3390/foods14162903
6. Cho, H.D.; Brownmiller, C.; Sorker, H.; Islam, S.; Lee, S.O. Sweetpotato leaves inhibit lipopolysaccharide-induced inflammation in RAW 264.7 macrophages via suppression of NF-κB signaling pathway. Foods 2021, 10, 2051. https://doi.org/10.3390/foods10092051
7. Lee, D.; Kim, J.; Baek, S.; Lee, J.W.; Lee, C.; Kang, K.S.; Shim, S.H. 1,3,5-tricaffeoylquinic acid from Ipomoea batatas vines induced ovarian cancer cell apoptosis and inhibited endothelial tube formation. Biomol. Ther. 2025, 33, 483–493. https://doi.org/10.4062/biomolther.2024.239
8. Luo, D.; Mu, T.; Sun, H. Profiling of phenolic acids and flavonoids in sweet potato (Ipomoea batatas L.) leaves and evaluation of their antioxidant and hypoglycemic activities. Food Biosci. 2021, 39, 100801. https://doi.org/10.1016/j.fbio.2020.100801
9. Zou, J.-Y.; Xu, Y.; Wang, X.-H.; Jiang, Q.; Zhu, X.-M. Improvement of constipation in leukemia patients undergoing chemotherapy using sweet potato. Cancer Nurs. 2016, 39, 181–186. https://doi.org/10.1097/NCC.0000000000000257
10. Nguyen, H.C.; Chen, C.-C.; Lin, K.-H.; Chao, P.-Y.; Lin, H.-H.; Huang, M.-Y. Bioactive compounds, antioxidants, and health benefits of sweet potato leaves. Molecules 2021, 26, 1820. https://doi.org/10.3390/molecules26071820
11. Jahurul, M.H.A.; Islam, S. Emerging Techniques for the Recovery of Bioactive Compounds from Sweet Potato Leaves (*Ipomoea batatas* (L.) Lam) and Their Functional Health Benefits. Food Bioeng. 2025, fbe2.70021. https://doi.org/10.1002/fbe2.70021
12. Hapke, K.L.; Azad, M.A.K.; Sultana, T.; Islam, S. Comparison of antioxidant, β-carotene, and phenolic levels between roots and leaves across three popular sweet potato (Ipomoea batatas L.) varieties. Agric. Food Secur. 2025, 13, 62. https://doi.org/10.1186/s40066-024-00516-x
13. Chen, C.M.; Li, S.C.; Lin, Y.L.; Hsu, C.Y.; Shieh, M.J.; Liu, J.F. Consumption of purple sweet potato leaves modulates human immune response: T-lymphocyte functions, lytic activity of natural killer cell and antibody production. World J. Gastroenterol. 2005, 11, 5777–5781. https://doi.org/10.3748/wjg.v11.i37.5777
14. Ishida, H.; Suzuno, H.; Sugiyama, N.; Innami, S.; Tadokoro, T.; Maekawa, A. Nutritive evaluation on chemical components of leaves, stalks and stems of sweet potatoes (Ipomoea batatas Poir). Food Chem. 2000, 68, 359–367. https://doi.org/10.1016/S0308-8146(99)00206-X
15. Johnson, M.; Pace, R.D. Sweet potato leaves: properties and synergistic interactions that promote health and prevent disease. Nutr. Rev. 2010, 68, 604–615. https://doi.org/10.1111/j.1753-4887.2010.00320.x

16. Krochmal-Marczak, B.; Cebulak, T.; Kapusta, I.; Oszmiański, J.; Kaszuba, J.; Żurek, N. The content of phenolic acids and flavonols in the leaves of nine varieties of sweet potatoes (Ipomoea batatas L.) depending on their development, grown in Central Europe. Molecules 2020, 25, 3473. https://doi.org/10.3390/molecules25153473
17. Nwosisi, S.; Nandwani, D.; Myles, E.L. Antiproliferative potential of sweetpotato in breast (BT549) and lung (A549) cancer cell lines. *BMC Complement. Med. Ther.* 2025, 25, 47. https://doi.org/10.1186/s12906-025-04770-9
18. Sultana, T.; Islam, S.; Azad, M.A.K.; Akanda, M.J.H.; Rahman, A.; Rahman, M.S. Phytochemical profiling and antimicrobial properties of various sweet potato (*Ipomoea batatas* L.) leaves assessed by RP-HPLC-DAD. *Foods* 2024, 13, 2787. https://doi.org/10.3390/foods13172787
19. Karna, P.; Gundala, S.R.; Gupta, M.V.; Shamsi, S.A.; Pace, R.D.; Yates, C.; Narayan, S.; Aneja, R. Polyphenol-rich sweet potato greens extract inhibits proliferation and induces apoptosis in prostate cancer cells in vitro and in vivo. *Carcinogenesis* 2011, 32, 1872–1880. https://doi.org/10.1093/carcin/bgr215
20. Xu, L.; Zeng, K.; Duan, Z.; Liu, J.; Zeng, Y.; Zhang, M.; Yang, Y.; Guo, Q.; Jin, Y.; Liu, W.; Guo, L. Screening potential antileukemia ingredients from sweet potato: Integration of metabolomics analysis, network pharmacology, and experimental validation. *Front. Nutr.* 2025, 12, 1518525. https://doi.org/10.3389/fnut.2025.1518525
21. Nakachi, S.; Tokeshi, A.; Takamatsu, R.; Arakaki, K.; Uehara, M.; Iguchi, A.; Taira, J.; Yoshimi, N. Abstract 840: The modifying effects of the extract from Okinawan sweet potato leaves in mouse colon carcinogenesis. *Cancer Res.* 2016, 76 (Suppl. 14), 840. https://doi.org/10.1158/1538-7445.AM2016-840
22. Vishnu, V.R.; Renjith, R.S.; Mukherjee, A.; Anil, S.R.; Sreekumar, J.; Jyothi, A.N. Comparative study on the chemical structure and in vitro antiproliferative activity of anthocyanins in purple root tubers and leaves of sweet potato (*Ipomoea batatas*). *J. Agric. Food Chem.* 2019, 67, 2467–2475. https://doi.org/10.1021/acs.jafc.8b05473
23. Zhang, N.; Ren, Y.; Xu, Y. From laboratory to clinic: Opportunities and challenges of functional food active ingredients in cancer therapy. *Front. Nutr.* 2025, 12, 1627949. https://doi.org/10.3389/fnut.2025.1627949
24. Meng, X.; Dong, T.; Li, Z.; Zhu, M. First systematic review of the last 30 years of research on sweetpotato: Elucidating the frontiers and hotspots. *Front. Plant Sci.* 2024, 15, 1428975. https://doi.org/10.3389/fpls.2024.1428975
25. Alam, M.K. A comprehensive review of sweet potato (*Ipomoea batatas* [L.] Lam): Revisiting the associated health benefits. *Trends Food Sci. Technol.* 2021, 115, 512–529. https://doi.org/10.1016/j.tifs.2021.07.001
26. Sun, H.; Mu, T.; Xi, L.; Zhang, M.; Chen, J. Sweet potato (*Ipomoea batatas* L.) leaves as nutritional and functional foods. *Food Chem.* 2014, 156, 380–389. https://doi.org/10.1016/j.foodchem.2014.01.079
27. Chang, V.H.-S.; Yang, D.H.-A.; Lin, H.-H.; Pearce, G.; Ryan, C.A.; Chen, Y.-C. IbACP, a sixteen-amino-acid peptide isolated from *Ipomoea batatas* leaves, induces carcinoma cell apoptosis. *Peptides* 2013, 47, 148–156. https://doi.org/10.1016/j.peptides.2013.02.005
28. Kurata, R.; Adachi, M.; Yamakawa, O.; Yoshimoto, M. Growth suppression of human cancer cells by polyphenolics from sweetpotato (*Ipomoea batatas* L.) leaves. *J. Agric. Food Chem.* 2007, 55, 185–190. https://doi.org/10.1021/jf0620259
29. Kang, H.-G.; Jeong, S.-H.; Cho, J.-H. Antimutagenic and anticarcinogenic effect of methanol extracts of sweetpotato (*Ipomea batata*s) leaves. Toxicol. Res. 2010, 26, 29–35. https://doi.org/10.5487/tr.2010.26.1.029
30. Chaachouay, N.; Zidane, L. Plant-derived natural products: A source for drug discovery

31. George, J.; Reddy, G.V.P.; Wadl, P.A.; Rutter, W.; Culbreath, J.; Lau, P.W.; Rashid, T.; Allan, M.C.; Johaningsmeier, S.D.; Nelson, A.M.; Wang, M.L.; Gubba, A.; Ling, K.-S.; Meng, Y.; Collins, D.J.; Ponniah, S.K.; Gowda, P.H. Sustainable sweetpotato production in the United States: Current status, challenges, and opportunities. *Agron. J.* 2024, 116, 630–660. https://doi.org/10.1002/agj2.21539
32. Laveriano-Santos, E.P.; López-Yerena, A.; Jaime-Rodríguez, C.; González-Coria, J.; Lamuela-Raventós, R.M.; Vallverdú-Queralt, A.; Romanyà, J.; Pérez, M. Sweet potato is not simply an abundant food crop: A comprehensive review of its phytochemical constituents, biological activities, and the effects of processing. Antioxidants 2022, 11, 1648. https://doi.org/10.3390/antiox11091648
33. Wang, S.; Nie, S.; Zhu, F. Chemical constituents and health effects of sweet potato. Food Res. Int. 2016, 89, 90–116. https://doi.org/10.1016/j.foodres.2016.08.032
34. Elgabry, R.M.; Sedeek, M.S.; Meselhy, K.M.; Fawzy, G.A. A review on the potential health benefits of sweet potato: Insights into its preclinical and clinical studies. Int. J. Food Sci. Technol. 2023, 58, 2866–2872. https://doi.org/10.1111/ijfs.16447
35. Gundala, S.R.; Yang, C.; Lakshminarayana, N.; Asif, G.; Gupta, M.V.; Shamsi, S.; Aneja, R. Polar biophenolics in sweet potato greens extract synergize to inhibit prostate cancer cell proliferation and in vivo tumor growth. Carcinogenesis 2013, 34, 2039–2049. https://doi.org/10.1093/carcin/bgt141
36. Islam, S. Sweetpotatoes [*Ipomoea batatas* (L.) Lam]: The super food of the next century? An intensive review on their potential as a sustainable and versatile food source for future generations. CyTA–J. Food 2024, 22, 1–26. https://doi.org/10.1080/19476337.2024.2397553
37. Zhang, Z.; Xu, G.; Ma, M.; Yang, J.; Liu, X. Dietary fiber intake reduces risk for gastric cancer: A meta-analysis. *Gastroenterology* **2013**, *145*, 113–120.e3. https://doi.org/10.1053/j.gastro.2013.04.001
38. Chinnadurai, R.K.; Khan, N.; Meghwanshi, G.K.; Ponne, S.; Althobiti, M.; Kumar, R. Current research status of anti-cancer peptides: Mechanism of action, production, and clinical applications. Biomed. Pharmacother. 2023, 164, 114996. https://doi.org/10.1016/j.biopha.2023.114996
39. Silva-Correa, C.R.; Hilario-Vargas, J.; Calderón-Peña, A.A.; Torre, V.E.V.-L.; Aspajo-Villalaz, C.L.; Bailon-Moscoso, N.; Romero-Benavides, J.C.; Herrera-Calderon, O.; Sagástegui-Guarniz, W.A.; Castañeda-Carranza, J.A.; Janampa-Castillo, W.E.; Cruzado-Razco, J.L. Protective effect of purple sweet potatoes (*Ipomoea batatas* L.) against rat breast cancer. Vet. World 2025, 18, 1137–1146. https://doi.org/10.14202/vetworld.2025.1137-1146
